# Selections and Abstracts

**Published:** 1905-04

**Authors:** 


### *SELECTIONS AND ABSTRACTS.*

### Roentgen Therapy: Its Sphere of Applicability.

The Roentgen rays have demonstrated a practical power to alter cells of low vitality and stimulate regenerative changes. They have proved themselves capable of effecting the cure of many chronic skin lesions in a remarkably short time, that have baffled other therapeutic measures. They have shown an analgesic power in severe neuralgias and rheumatisms, which in most cases is combined with alterative effects that result in permanent relief and restoration of function. They have demonstrated an inhibitory action over the growth of malignant cells that has amounted to a cure in epitheliomas and superficial growths, while for want of time to prove their permanency, the remarkable effects seen in graver lesions can be termed as yet only an inhibitory action. In tubercular manifestations, especially in lupus and tubercular adenitis, they have been found the most effective therapeutic agent known. Besides all these effects they have proved equally efficient in their action upon non-malignant growths.

It is because their action is stimulant and alterative that they have found so wide a field of application. Whenever they can be applied to stimulate altered metabolism or act upon pathologic cells of low vitality, they have proved effective agents in restoring normal conditions.

This demonstrated power to produce beneficial effects, in conditions that have previously been hopeless or have become chronic, makes it essential that every conscientious practitioner should appreciate the condition in which they may be relied upon to aid in treatment, either primarily or as supplementary to operation or other methods of treatment.

For convenience the classes of cases may be divided into non-malignant skin lesions, including tubercular and glandular lesions, neuralgias, neurites, and rheumatic conditions and superficial and deep malignant growths.

The varied and apparently antagonistic effects which can be produced by the proper dosage of this therapeutic agent finds no better illustration than in its application to *hursitis* and *alopecia areata.* Here the alterative action as well as the stimulative must be taken into consideration. The highly specialized hair follicle can be destroyed without injury to the surrounding normal structures and the hair removed, or the dose can be regulated to produce only a stimulant action and thus restore the normal vitality. According to the dose given the hair is removed permanently, or, if the follicle is not totally destroyed, the hair can be made to grow again. The depilatory action of this agent is very valuable in the treatment of other dermic lesions, as *sycosis* in which the disease has invaded the hair follicles and all hair must be removed before a cure can be effected. These lesions yield rapidly to treatment, and the cure can be permanent without the total destruction of the hair. This condition will serve to illustrate its application to all lesions where the hair follicles are involved.

Severe *acne,* particularly the pustular type, is especially amenable to treatment by this method. The pustules break and heal with less scar formation than after curetting, while chronic inveterate cases yield rapidly to this stimulant and alterative.

The action of Roentgen rays upon skin lesions is apparently more thorough than that of ointments and lotions; it seems to reach deeper and produce alterations and destructions of the deep-lying pathologic tissues. This is illustrated forcibly in its action upon *eczema and psoriasis.* In eczema, whether of the dry or moist form, and in cases of even five and twelve years' standing, the treatment has proved remarkably efficient. The same is true of psoriasis, of which a severe case yielded permanent results, though the treatment had to be vigorous and extended over a number of months. In these cases of local manifestation of what is apparently a disease due to systemtic causes, there can of course be no guarantee against the recurrence of the disease in other portions of the body.

The remarkable absence of scar tissue in the results following this treatment, even where there has been loss of substance, render it particularly valuable from the cosmetic standpoint. This effect in removing scar tissue is demonstrated more thoroughly in its effect upon *keloids*, which yield readily to this agent.

In speaking of tubercular manifestations, *lupus* or tubercular ulceration claims attention because of the great difficulty in producing a cure by other known means, and the almost specific action which the Roentgen ray has shown. No tubercular skin lesion has failed to react to appropriate dosage in my hands, and this is true of every one who applies this agent judiciously. Many other tubercular lesions have shown a similar readiness to yield to this therapy. Tubercular adenitis and simple adenitis of the cervical lymphatics, even when due to, and following, exanthemata, yield rapidly to this agent, without the necessity for fixation dressings or even simple incision if they are taken early before suppuration has ensued. The great advantage of early treatment in these conditions, where such excellent results can be obtained without disfiguring the patient, are very manifest. The treatment can be undertaken earlier and carried out much more thoroughly than when fixation collars are to be worn or operation consented to. Other tubercular lesions as synovitis, tenosynovitis, orchitis and arthrites of small joints have been very beneficially influenced, but the cures can not be attributed wholly to this agent, for with it, as in all other conditions, the patient demands the continuous care of the family physician to keep the physical condition and recuperative power as high as possible.

One of the most remarkable effects which this agent has produced is in the relief of pain. It was first noted in cases of malignant disease, where its palliative action was most valuable. The observation of this action led the author to employ it in a case of *acute neuralgia* following influenza. Five short treatments gave relief immediately and resulted in a permanent cure. Encouraged by this result it was applied in *chronic face neuralgia* of six months' duration, in a patient who had been the subject of neuralgia for twelve years, with relief and finally a cure that has lasted two years. It has also relieved a case of *migraine* in which frequently recurring attacks had persisted for twelve years, so that the patient has not had an attack for fourteen months.

A case of severe *tic douloureux,* in which an acute attack had resisted the best treatment for six weeks, was relieved almost immediately of pain and finally cured, and has remained well for the past eight months. Where, however, a *true neuritis*, with spots of local anesthesia, has developed, nothing can be promised but temporary relief from pain, while the chances of complete restoration and recovery depend largely on the chronicity and severity of the lesion.

As might be expected the pain of *rheumatism* yields to the analgesic influence of this agent, while the pathologic inflammatory process is beneficially influenced in most cases by its alterative action. The results in *chronic rheumatoid arthritis* are surprising, but are comparable to those it produces in other chronic conditions. The results in some cases of *arthritis deformans* are truly remarkable.

This brief review of the non-malignant conditions does not include the purely medicinal cases in which very valuable results have been produced, as in Addison's disease, tubercular peritonitis, and the various forms of goiter, as too few cases have been reported upon which to base any valuable conclusions.

In dealing with the application of this remedy to malignant disease, other factors than its demonstrable effects must be taken into consideration, because of the known tendency of malignant disease to recur, and the difficulties that lie in the way of establishing the permanency of a cure.

Up to the present time we can only say that an inhibitory action upon the growth and development of malignant disease has been demonstrated, as it is too early to prove that any case has been cured. Under these conditions it is absolutely essential to scientific judgment that in all cases, where the patient's life would otherwise be jeopardized, and where operation is permissible, operation must precede Roentgen treatment. The patient must be given all the possible chances of the best known-methods. It is, however, equally certain that the demonstrated power of the Roentgen rays over the growth of malignant cells makes it equally essential that every patient be given Roentgen treatment as well, or his chances for recovery will be lessened.

Superficial lesions, such as *epitheliomas* and *rodent ulcers*, that do not necessarily threaten the life of the patient can be treated with advantage primarily by the Roentgen method, because they can be effectively destroyed, and with a resulting scar that is not apprecia-ble or disfiguring. Scirrhus of the breast is a condition in which surgical teaching the result of pathologic finding seem to contra-indicate operative intervention. This condition yields rapidly to Roentgen treatment.

The rapid recurrences which generally follow operations upon *sarcomata* and their distribution into regions remote from the orig-inal lesion are facts which, combined with the efficiency noted in the Roentgen treatment, especially where there has been no opera-tive interference, would seem to indicate a tentative course of Roentgen treatment in these cases, to be followed by operation if acute conditions demand it.

The only other exception to early radical surgical removal of the malignant disease is in recurrences or massive growths that arise too late for any treatment. Here the removal of the larger masses facilitates the effective Roentgen treatment, and renders, even in hopeless cases, its palliative action of the greatest benefit.

The demonstrated effective inhibitory and destructive influence of this agent over malignant disease makes it essential as a supple-ment to every operation for the radical relief from malignant dis-ease. It then can attack any remaining foci of malignant disease that have escaped the operator, while they are devitalized as the result of the operative trauma and before they have had time to engraft themselves upon the tissue of the patient. While the mor-tality of malignant disease remains so high after operation, it is essential that every means at command be employed in combating it. An agent which has demonstrated such power over malignant cellular activity can not be neglected without seriously impairing the patient's chances. The post-operative treatment in no way interferes with the patient's recovery; in fact, hastens it. Treat-ment can be given effectively through the dressings, and if no irri-tating antiseptic has been employed it facilitates and hastens the healing. As a palliative in hopelessly inoperable cases there is nothing which is more efficient than the Roentgen rays in reliev-ing the patient's suffering and rendering life tolerable; while many remarkable cases have demonstrated that it is impossible to say how much such treatment may lengthen life.

It must be clearly understood that these results are attainable, even in superficial benign lesions, only when the proper dose is given in proper time, quality and quantity to effect a lesion to which its quality is particularly adopted. Quinine might as well be given to cure syphilis, or mercury to cure malaria, as to use the wrong quality of Roentgen discharge in treating any lesion. The quality and quantity can only be produced with the development of special technique, and the acquirement of clinical experience in adapting them to the lesion in hand.

This agent is, like all others, incapable of producing results unless properly employed, and efficiency is due as much to the way in which it is employed as to the agent.—*Charles Lester Leonard, President of the Roentgen Ray Society, Philadelphia, in Monthly Cyclopedia.*

---

### MASSAGE VS. OSTEOPATHY.

BY MR. A. ABERG, Denver, Colo.

It is a well-known fact that physicians all over the country are particularly averse to the claims and methods of the so-called osteopaths, who, taking their place among the quack practitioners of the healing art, fill the cheap newspapers with flaming advertisements of what they have done, and of what they can do. They are now found in every community. That their claims of cures are specious, aside from the little good they may do in certain diseases along the line of massage, there are few who will undertake to deny. Still they flourish, and in most instances to the direct detriment of the skilled and legitimate doctor. They know little or nothing of anatomy, and little or nothing of genuine massage, as it is taught in the schools of this country and Europe.

Without going into any extended description of the methods used by these unskilled osteopaths, many of whom dropped the plow or shovel to take up their "profession," it will suffice to say that their mode of treatment is nothing more than the lowest grade of unskilled massage. And, in fact, it is oftentimes of so violent a character that the poor victim is left permanently injured. Several instances of this kind can be cited in Denver. A delicate child is taken to one of these osteopaths, who proceeds at once to make a diagnosis. He finds a bone out of place, one or two of the vertebrae perhaps, and he goes to work with his grubber's strength to correct the evil. The little victim is twisted, mauled, rubbed and stretched in a most brutal fashion, much as if he or she were paying penance in the inquisition of olden times. But it is all necessary to get the recalcitrant vertebra back into place. Just when it left its snug and accustomed place in the spinal column to wander over in the region of the epigastrium, no one can ever tell, but the muscular "professor" finally drives it back, complainingly perhaps, to its duty in the median line. If the child survives the ordeal, he or she promptly gets well, more than likely as an acceptance of the horn of the dilemma farthest removed from falling into the hands of the muscular "professor" again.

Notwithstanding these well-known facts, physicians do nothing to stay the progress of this nefarious practice beyond verbally condemning it occasionally. As the guardians of public health and the public welfare generally, they should do more. They should use one of the best weapons in the world against the osteopaths—massage.

There are in nearly every city in the United States one or more skilled and qualified massage operators, who have learned their profession in reputable and recognized schools in this country or abroad. They look to the doctors for work, to be done under the doctor's supervision, but many of them look in vain. It is quite true that massage, as a profession, has been grossly abused because of inadequate protection, and that physicians have therefore not always obtained the results that they had a right to expect, but that should not deter the doctor from employing this legitimate and recognized therapeutic agent when a skilled operator is so easy to secure. The physician should decide if his patient could be benefited by a thorough course of scientific massage, and then should recommend a certain operator to him, who would give the massage under the physician's directions. In this way the physician would retain his patient, and prevent him from falling into the hands of unskilled osteopaths.

Before employing or recommending a massage operator the physician should always make the fullest investigation in regard to the operator's ability and qualifications, as well as to his or her general character. As the newspaper men say, "it is results that count," and in this way the physician can count on results. Then, if he be fully qualified in massage, he should give minute directions, a prescription I might say, as he would for the combination of drugs that may accompany the manipulations. This is done by many physicians in other cities, and I may say by a few in Denver.

Those physicians who have not had the advantage of a course in massage can go to any qualified operator and enjoy a trial treatment or two, which will not only demonstrate what the operator can do, but give him a fair idea of the movements and their uses for his future recommendations.

In reading any text-book on medicine or surgery we are constantly reminded that massage is a valuable therapeutic agent in many ailments of the human body. In reading any cheap newspaper we are daily reminded that the osteopaths are abroad in the land, taking advantage of this fact to ply their vocation of rounding up the wandering bones in the human body, to the detriment of the regular physician. The skilled massage operator is a helpmeet of the physician, occupying a legitimate place at the right hand of the physician, while the osteopath is a catcher of suckers through a loop-hole in the law.

Professors in medical colleges, in their lectures, will tell the students that massage should be employed in this or that disease, and that it is of recognized value. Still the same professor who so ardently recommends massage in his lectures, perhaps never recommended it in his private practice, and nine times out of ten he will freely confess, if approached on the subject, that he knows nothing whatever about the methods of applying this kind of treatment.

On the European continent massage is held in high regard. The physicians not only employ the services of male and female operators, but many prominent physicians practice the art themselves as a specialty.

In many universities of Europe a chair for teaching massage to medical students is regularly provided. These men, when they leave school to begin the practice of medicine, are also practical massage operators. There the bone-replacing osteopaths are unknown. The deduction is plain. The way to stamp out the osteopathic fad is to lift massage to the place where it by right belongs, and this can only be done by recognizing and employing well qualified massage operators. In this way the laity will be educated to distinguish between scientific massage and osteopathic quackery.

A therapeutic agent that has such able advocates as S. Weir Mitchell in this country, and Lorenz, in Europe, as well as thousands of the most brilliant physicians all over the world, should be studied by every medical man, and given the consideration and place it so well deserves. And in doing this the physician would effectually block the progress of one of the most specious fads now in existence.—*Colorado Medical Journal.*

---

### GENERAL VIBRATORY MASSAGE.

BY CARL SANDZEN, A.M., M.D.
Kansas City, Mo.

General massage as a therapeutic agent has not been known very long in our country. The first physician who fully understood and proclaimed its great value was Weir Mitchell, who has shown how in so-called forced feeding or in any kind of abundant nutrition the body can not receive the full benefit from the treatment without general massage. He tried it with success and other physicians have followed his teachings with the same good results.

One condition that should not be overlooked is that general massage ought to be properly given, otherwise its value is very small. Every physician who has worked along this line has certainly had many discouraging experiences in his dealings with poorly educated, ignorant masseurs and masseuses. This has led to the use of mechanical devices for the application of general massage. I do not need to repeat here how and why vibrations can give sufficient exercise. We accept it as a given fact. The only purpose of this article is to explain how it should be given to get the best results. The time for treatment must be chosen to suit different cases with which we have to deal, e. g., in a case of insomnia, give the treatment as late in the day as possible. If given only as exercise, however, as a substitute for walking, riding, etc., I consider the morning the best time.

The patient should be dressed in underclothing only, with the shoes removed, a soft union-suit is the best dress of all. Place the patient in a recumbent position on an operating table or chair, if the massage is given in the physician's office, otherwise on any suitable table or couch. The head should be slightly elevated with a pillow, the legs bent at the knee joints, with the feet somewhat apart and the whole sole placed on a support. This position is the best on account of the relaxation it gives to the muscles of the abdomen and the extremities. All massage movements should be given from the periphery to the center, viz, the heart. Every treatment in general massage ought, therefore, to begin with the feet or hands, usually the feet. Move the vibratode with increasing pressure on the foot from toe to heel or on the hand from the finger-ends to the thenar eminence. Proceed to the calf of the leg and thereafter to the thigh, or, if you choose, treat the upper extremities first, giving to the stroke the length of the whole arm. After the extremities, comes the treatment of the abdomen. Begin the treatment on the right side, somewhat below the cecal region, going upward along the colon ascendens, then following the colon transversum, and at last, colon descendens and flexura sigmoidea. Begin every course anew over the point of exit and finish with vibration from side to side across the abdomen with the purpose of stimulating the small intestines. About five minutes is enough for the abdomen in general massage. Vibrate on the chest either from side to side or up and down.

When the front side is finished, turn the patient on his stomach and treat the back. Move the applicator up and down on each side, up the spine, and from the middle line to the posterior axillary line on each side. On the gluteal muscles, one should use very powerful vibrations and should move the vibratode in the direction of the different muscle-layers, using a good deal of pressure. This constitutes what we call a general treatment, as we never include the head, except in special cases which require vibrations on the head or neck.

No seance should last over twenty minutes, or, at the most, twenty-five minutes, the time being divided according to the operator's best judgment. Large muscle groups require larger and more powerful treatment than smaller ones, etc.

General massage should be given every day if possible, otherwise at least every other day. It is very convenient to have two vibrators, one on each side of the operating table, as it saves a good deal of trouble in turning your patient or moving the apparatus. It is also very good economy to use two machines, as excessive use will destroy (by running hot, etc.,) even the best apparatus.—*Detroit Medical Journal.*

---

### EXPECTANT TREATMENT.

BY A. JACOBI, M.D., LL.D.
New York.

When fifty years ago I graduated in medicine, Samuel Hahnemann had been dead only eight years. His principal influence on therapeutics was not attained by his rejection of blood-letting, or by his *similia similibus* theory, but by his potential dosing, by which he pretended to prove that a drug would exhibit its remedial power only when no trace of it could even be found in the medicine a patient was furnished. In this way it was demonstrated, not so much by him as in spite of him, that the sick might recover without medicine. At the same time, based upon the brilliant achievements of Broussais and the whole French medicine of the first forty years of this century, the Vienna School, under Rokitansky, confined its research and its teaching to pathologic anatomy and diagnosis. For in the opinion of Rokitansky, medical science was limited to dead-house studies, and the greatest Vienna clinicin, Skoda, pronounced the axiom that a disease could be diagnosticated, defined, and comprehended, but there were no

---

*Editor's Note.* Dr. Jacobi's paper on Expectant Treatment contains so many important truths, that I think I am conferring a favor upon my readers in reprinting it. For some reason or other the paper did not get the attention it deserved when it first appeared some three years ago.

means to cure it. At the same time, to give a single instance only, Dietl treated 750 patients suffering from pneumonia without venesection and drugs, with a mortality of only 69, that is, less than 10 per cent., and thereby proved that the alleged results of what was called homeopathy in those times were fallacies. He also confirmed the existence of what Hippocrates and hundreds of his followers knew as *vis medicatrix naturae;* that is, the inherent tendency of many diseases, when not interfered with, to terminate in recovery. Even Wunderlich, of Leipzig, in his first period proclaimed that medicine should be science but not art. All this was proclaimed in spite of the teachings of one of the members of the Vienna circle, Hebra, who demonstrated daily that many local diseases hitherto considered incurable, could be cured by, and absolutely required, local treatment.

That nihilism was based on the harm which was constantly done by the polypharmacy of former practices, and the nastiness of their theriacs and other compounds, and partly on the lack of knowledge of reliable medicines and of their action. No nihilism, however, could after a while withstand the growing influence of pharmacology and of systematic experimentation; but what has been called expectancy has taken its place. Expectancy and expectant treatment mean, if anything, the method of observing the course of an illness and the indications for treatment, with the express understanding that treatment with drugs should be avoided except under the most urgent necessity recognizable even to the average observer.

The somewhat fragmentary remarks I shall make are written for the purpose of justifying treatment, and particularly medicinal treatment, to a greater extent than writers appear anxious to permit—to no greater extent, however, than far-sighted practitioners justify by their practice. What I shall have to say I want to be understood as a plea for timely and energetic medication, and for the suppression of the very term of expectancy as needless and misleading.

Expectancy in treatment has its well-defined causes:

(1) Reliance on Nature. By all means rely on her, but do not forget that you and your tools and your remedies are also part of

Nature. The reliance on the power of Nature in contradistinction to the effort of man's doing, is one of the axioms due to a trinity of modesty, ignorance and laziness. *Natura sanat, medicus curat* is on everybody's lips, medical man's and layman's, the latter's mainly, when in health he snubs and ridicules the doctor, while he sends for him in hot haste when falling sick. What the doctor is credited with, perhaps, is that he takes care of the patient, and sees to it that Nature may perform its work.

Before a Philadelphia audience, some years ago, the same subject was discussed; a few notes of what was there submitted may be admitted here. Here is an example: "Nature was kind enough to so reduce hemoglobin as to give you an opportunity to render a girl a service by feeding her on iron. Nature saves her by kindly giving her a stomach and a viscera and lymph apparatus to digest your iron; that is why it is not you that has anything to do with her recovery. Oh, no. But then, there are other cases of chlorosis where Nature supinely furnished congenitally small arteries and an incurable chlorosis. Most complacently Nature looks on; man, however—that is you—is blamed for not making progress against impossibilities. You think you heal a man poisoned by plasmodia by giving him quinine, arsenic, or ergot; again a mistake, for it is Nature that raises cinchona trees and furnishes digestion; maybe quite often for that reason Nature is credited with the good result, and the patient feels justified in not paying the doctor either thanks or debts. You think you save a man by cutting down on an appendicitis or a liver abscess. Far from it, you are only the scavenger, but Nature forms exudations and adhesions. You keep skin and table and tools aseptic, and think you did a praiseworthy thing in the way of prevention. What of it? Nature permitted man to invent soap and sublimate and created a healthy cell proliferation. You find a man in the gutter with a sunstroke, kindly donated by maternal Nature; you work over him for hours with ice and stimulants and friction; no thanks to you, it is 'Nature' that empties his cerebral blood-vessels, eliminates toxins, and restores him. You are expected to and believe you heal a fracture; that is what you can not do; Nature does it. Can you make new cells? Can you form callus? What you can accomplish is to adapt the ends of the bones and to appear as defendant in a suit for malpractice." You say all this is farcical? So it is, but the absurdity is not mine. If there be anything insipid in man's so-called reasoning, it is this unmeaning fighting about words, this wiseacre talk about the relations of "Nature" and doctor.

Nature does not kill and does not cure. If there were consciousness in her she would feel indifferent about what she is, viz., mere evolution. With her sunshine she grows harvests and sunstrokes; her moonshine favors lovers and burglars alike; her rain feeds men and drowns them; her wind fertilizes trees and destroys the abodes of a thousand people. Nature is a Mauser bullet; stand in its way and it hits you, dodge and you are saved. It makes no difference to Nature.

In Nature a diphtheria bacillus has its democratic rights and democratic duties like George Washington—that is why it could kill him. She has no predilections and no reasoning; she is simply cause and effect. That is why she can be guided and misguided by you, by engineers; and why the logical mind or the whim of man and the logical necessities of Nature are engaged in a constant strife for superiority. In matters of health and life the medical *homo sapiens* utilizes or combats the doings of Nature. By caring he cures.

Unfortunately, or fortunately, curing—"*curare*"—has long lost its literal meaning. Curing is healing.

(2) The practitioner has no clear indications and resorts to expectant treatment, which is merely no treatment, but an idle looking on, because of his insufficient diagnosis and prognosis.

Ignorance makes cowards. The nature of a disease is learned, besides its symptomatology, by its pathologic anatomy, in the same way that physiology must be founded on gross and histologic anatomy.

Unless the practitioner is a good diagnostician and observer—for no accurate prognosis is possible without a large number of close observations of the full course of morbid processes—he will never be a good and reliable therapeutist.

If a man be ignorant and careful and keeps his hands off, he will never do wilful harm to his patient, and will let him get well or die undisturbed. But not knowing why and how, he will never benefit him.

Unfortunately for us, most ignorant men are not modest nor cautious; unfortunately, also, most of our work is brain work; those without brains, therefore, should not be among us. That is why neither our calculations nor our results can be satisfactory always. Those of us who work with hands and brains equally, or with hands preferably; that is, the operative surgeons—are better off. Their aim is simple, their disease local, their treatment direct; even their diagnosis is confirmed or refuted by their autopsy on the living, and their medicine simple and effective. Iron in their hands is a different weapon from that on which we read tracts and books innumerable without being sure that we understand its action.

(3) The physician is not sure of the action of his medicines; not even specifics, such as quinine, salicylic acid, antisyphilitics, and antitoxins are absolutely certain in their effects. Even emetics and purgatives act differently in different persons; so do narcotics in the mentally sound, particularly, however, in those unsound. Cardiac tonics and stimulants exhibit the same difficulties; their doses can not be determined by ages or by body weights; the young require very much greater relative doses. Likewise, in the hands of the very best observers, large numbers of cases and observations are required for trustworthy results, and the published records of observers of unequal acumen or experience are far from being reliable guides in different seasons, localities and climates. Not infrequently the difficulties are increased by the condition of the drugs; digitalis, pomegranite, ergot, pilocarpus, are of unequal value in different countries, and are changed by age or manipulation. Tinctures and solid extracts of many herbs change their nature and activity. Adulterations are frequent. That is why the raw material and many preparations of many herbs are unreliable. Even simple bodies like milk-sugar are adulterated, and are far from serving the purposes they are used for.

Let me give you a few instances only, of expectant treatment of my own. I want to prove the undefined nature of the boundary line of what may be taken for justifiable delay, but is irresolute ignorance, or cowardice.

It was forty years ago—I kept the notes of the case—that a young man complained to me of pain and swelling in his right side above the liver. He was treated expectantly, in the hope that if an abscess would form it would point externally. Nothing was done. One day the abscess broke into the pleural cavity. My patient died, but his case is one of those that, after nearly half a century, turns up in an occasional sleepless night.

It is not the only one of its kind; nor shall I ever be less disturbed by another piece of expectant treatment.

Forty-six years ago I was at least as young, ignorant, irresolute, and helpless as to-day. There was a woman—I know her face to-day, for I have seen it a thousand times since—who died in her first confinement; so did her child, because I killed time and opportunities by bothering with the forceps while I should have saved two lives by Cæsarean section.

[We fully agree with the venerable author in his arraignment of expectant treatment, which is frequently but a synonym of laziness or irresoluteness. But there is, of course, another view to be taken of this matter. For every case lost by "expectant" treatment there are probably two or more cases spoiled by over-zealous meddlesomeness. To wait or to act—that is the oft-repeated question in medicine. Unfortunately, no text-book and no paper can give us a dogmatic answer.—EDITOR.]

Having laid myself open to your criticisms, I am egotistic enough to speak of the expectant mistakes you are making, or many of you, or a very few of you.

What some one here present may be doing constantly is as follows:

A snoring mouth-breather is treated expectantly—that is, not at all. No regular nasal irrigations are made that most infants and children should be subjected to—indeed, it is of more importance to wash the dirty little nose inside than outside. Big adenoids are not removed, large tonsils are not resected.

What you by your "expectant treatment" cause and are responsible for is, at least, insufficient aeration, pallor, defective development of the chest, and chronic indigestion with its lifelong results, as also the constant danger of coccus or bacillus invasions. Microbes may be counted by the millions and rendered harmless when merely deposited on the mucous membranes of either nose or pharynx, as long as the epithelia are in a healthy condition ; but the undisturbed presence of catarrh, adenoids or chronic pharyngitis, which invite or facilitate microbe invasion, is a direct *causa proxima* for tuberculosis, diphtheria, rheumatism and meningitis.

Suppose you saw a child in a clean, healthy house and family, with slight pharyngitis, perhaps only on one side. You know that when a pharyngitis is found on one side exclusively that can be the result only either of a local infection or a trauma. You know that; the parents and children do not. Still the child appears to be well, with little or no rise in temperature. The doctor is apt to say that he hopes it is nothing, and as that fully agrees with the hopes of the mother, the latter authority thinks well of the doctor because he is of her own so-called thinking. Twelve or twenty-four hours later he is told that there are white spots in the throat, and soon he knows that he has lost the same number of hours. Though the case may not be one of bacillus diphtheriæ, which you can not learn until six or ten more hours have passed by, but of the coccus variety, the latter, though benevolently neglected by our conservative boards of health, are infectious and contagious and quite often the source of great danger.

Instead of giving an otherwise innocent dose of ½ or 1 milligram of corrosive sublimate in a teaspoonful of water every hour or half hour, and preventing the full development of the microbic intoxication, you prefer to make no diagnosis, to approve of a mother's prognosis, and to show your respect to Nature by Micawberic indifference called "expectant" treatment.

In every attack of diphtheria, apparently mild or severe, you have to expect or to fear heartfailure—the result of toxins causing parenchymatous degeneration of the muscle ; you know it may come, aye, it will come. You treat the baby as if that would never happen. Every death that will occur, unless you have given ample doses, large doses, of an alcoholic beverage—first quality—no fusel oil whisky, which is a depressing poison—should be laid at your door and attributed to your "expectant treatment."

Why do your cases of pneumonia prove fatal? Some of them—a few only—perish from the paralyzing influence of the infection, many of the accompanying or complicating nephritis, most from heart failure. If the latter be controllable, the responsibility is ours. We know a lobar pneumonia will seldom terminate its exudative period in a few days, usually in six or seven the duration of a lobular pneumonia can not be calculated or estimated, and though it does not kill the first day or two, its mortality is greater.

In both we know that from day to day inanition must grow, the heart becomes feebler, failure be more imminent and death more sure. Expectant treatment waits until these symptoms, which you are sure are preparing, really come along. When your enemy is inside, you try to close the door behind him and begin your race with death when it is too late. Then, when there is no longer a circulation, you hope in vain for the absorption of your stimulants, either in the stomach or the rectum or the subcutaneous tissue—strychnine, ether, camphor, hot injections, whiskey—in vain.

The post mortem verdict may vary—I know of what I speak. "The doctors did everything they could." "When they saw the baby was dying they stood by us to the last." "We called a consultant, who knew no more than the rest, for, indeed, the darling died almost as soon as he came."

The facts of the case are these: That many a case of pneumonia dies in spite of your best directed efforts, but many more of your expectancy. You have no right to expect your case to proceed differently from or better than others. From day to day the heart must and will lose strength; *when* this persistent loss will terminate, you do not know; it is your duty to prevent what, without your interference, will certainly happen. Begin your stimulation (which at first should not be alcoholic) coupled with judicious feeding and care of the digestion, at an early period of the illness, and do not forget one—shall I say *the*?—principal object in view.

Listen, if you please. To have died is no calamity to the dead; mourning for the dead may be an easy matter for many; to many, however, that remain behind, it is a canker that grows in their hearts forever. Still the greatest calamity of all is long invalidism; much of this may be avoided by attending to the heart at once. Those of us who for the last fifteen or twenty years have followed the studies connected with the myocardium, are aware of the many dangers, shortening or embittering existence, that are wrought by pathologic changes of the heart muscle, formerly not noticed or not appreciated.

Permit me to quote a few lines from a former paper*: "Should medication begin when collapse is setting it, or has occurred? This systematic procrastination is parallel to giving nourishment when inanition is complete, and not before. When the donkey of the gospel disappears in a ditch on Sunday, make haste to pull him out on Monday. Allow the child to drown in your well, and be sure to cover it up—the well, I mean—on the day of the funeral. Build earthworks quickly when the enemy is in your camp. That is the same theory and practice according to which antitoxin is injected on the fifth day instead of the first, alcohol is refused in sepsis, digitalis in full doses in dilation and weakening of the heart, ice and opium in peritonitis, morphine in alcoholic delirium, or venesection in acute overdistention of the right ventricle."

Extend your consistency in a mistaken course of acting, or rather, abstaining from action, and you are no longer within the domain of art based on science, but approach the dangerous border line of the Christian Science criminality.

To those measures which are required to cover vital indications, I do not even allude. I do not choose to suppose that the attempts at saving life in accidents could ever be omitted. To that class, like the cutting of the rope around the neck of a suspended person, belong tracheotomy, or intubation in cases of suffocation from internal causes; artificial respiration by mechanical means and electrization of the phrenic nerve, transfusions, and salt-water infusions; also the administration of antidotes to poisons, also the subcutaneous dosing with stimulants.

Next in order are operations which should be understood and practiced by every pratitioner; besides tracheotomy and intubation, paracentesis in all cases of pleural, abdominal, cerebrospinal,

*Philadelphia Medical Journal, December 17, 1898.

and intradural effusions; herniotomy and operations for the relief of immediate dangers occasioned by osteomvelitis and neoplasms. In all of such cases the indication may be so urgent that the question is no longer one of the treatment of a disease, but the immediate saving of an endangered life.

There are other indications almost equally urgent. Prevent chronicity and sequels of a disease by timely interference, and by shortening its course. Though whooping-cough is a self-limited disease, it should be mitigated or cut short, for every day of its duration brings with it the possibility of hemorrhage, bronchopneumonia, and possibly of tuberculosis.

Avoid amyloid degeneration by attending to abscesses and necroses; the relapses of malarial fevers by giving quinine after the disappearance of the attacks, for a month or two in lengthening intervals. Wherever rheumatic polyarthritis has once existed, with or without endocarditis, see to it that your patient may never be without doses of salicylates on hand, which are to be taken as soon as the slightest pain will have appeared. By resorting to such measures you will have ample reason to rejoice over many cardiac diseases avoided and deaths averted. Take advantage of the quiescence of the appendix and save the person's life by means of an operation, the time for which you can select, and the prognosis of which, when thus performed, is almost certain to be of the best instead of standing expectantly by and looking forward to an acute attack that will take him off, operation or no operation.

I am aware that I have not given you anything new, no new clue to diagnosis, no new microbe, no new drug. But I thought I might lay before the young men among you some of the lessons a long life taught me and impressed upon me. During these fifty years there were few days in which I was not called upon either to cure or prevent, the performance of which duties is frequently invisible and as unpretentious as it may be made useful.

These lessons are not exclusively my own. They were conveyed by and compared with what I could learn from old and young. In my contact with the former I learned what to do and not to do; the latter have often impressed me with their enthusiasm and incited mutual criticism. As I was ever of the opinion that I had a right to claim as much youth and ignorance as they cheerfully displayed, I was always ready to be taught and corrected, and could afford to do so because I need not give up the direction given my life by the compass controlling modern medicine. The impetus which gave medicine its biologic character dates from the first of my medical studies. That character appeared at first to lead our path away from the goal of all medicine; viz., the prevention and cure of disease, and improvement of the mental and physical condition of man and mankind; but soon proved the correctness of Benjamin Franklin's saying that no science was good for anything unless proved beneficent to mankind. In that sense every progress in the science of medicine contributed to the art of healing and aided in helping to create the era of therapy in which we now live. To benefit a few among you whose professional career may not yet have attained or firmly planted its aim or ideal, I have written these few pages both as a review and as a platform. That platform should be:

In order to obtain indications for treatment, make a diagnosis. That art is becoming both more accessible and, through honest and hard work, more easy with the aid of modern methods. Remember that most diseases have, indeed, a tendency to spontaneous recovery, but also that recovery is not always complete and that invalidism should not be invited through neglect of treatment. Complications are possible as long as an illness lasts, and with every day cut short, the dangers of an otherwise typical disease are diminished.

The experience of old men in the profession who claim they employ less drugs with advancing years, means sometimes either an inability to master new methods of diagnosis, or the knowledge of new remedies. There is no one treatment for a disease adapted to every patient. There is no such thing as a uniform treatment for pneumonia, or for typhoid fever, or, even in spite of antitoxins, for diphtheria or tetanus. We should not try to treat the name of an illness, but the patient.—*Critic and Guide.*

